# Serum alkaline phosphatase levels correlate with long-term mortality solely in peritoneal dialysis patients with residual renal function

**DOI:** 10.1080/0886022X.2019.1646662

**Published:** 2019-08-14

**Authors:** Xiaojiang Zhan, Yuting Yang, Yanbing Chen, Xin Wei, Jun Xiao, Li Zhang, Caixia Yan, Panlin Qiu, Siyi Liu, Qinglan Hu, Qinkai Chen, Yu Wang

**Affiliations:** Department of Nephrology, The First Affiliated Hospital of Nanchang University, Nanchang, China

**Keywords:** Alkaline phosphatase, residual renal function, long-term mortality, peritoneal dialysis

## Abstract

**Introduction:** Increased serum alkaline phosphatase (ALP) is predictive of a higher mortality in patients with end-stage renal disease. However, it remains unknown whether residual renal function (RRF) influences the outcome-association of serum ALP among peritoneal dialysis (PD) patients.

**Methods:** A total of 650 incident PD patients receiving PD catheter implantation in an institute between 1 November 2005 and 28 February 2017 were retrospectively enrolled. These patients were divided into groups with and without RRF (RRF and non-RRF groups) and those with serum ALP levels in tertiles. The Kaplan–Meier method and multivariate Cox proportional hazard models were used to analyze their outcomes based on RRF and serum ALP levels.

**Results:** These 650 patients had a mean age of 49.4 ± 14.0 years old, their median ALP level was 74 U/L (interquartile range (IQR): 59–98). After 28-month (IQR: 14–41) follow-up, 80 patients in RRF group and 40 patients in non-RRF group died. PD patients with the highest serum ALP tertile had significant lower survival (*p* = .014), when compared to other patients in the RRF group. However, this relationship was not observed in patients in the non-RRF group. After multivariate adjustment, in the RRF group, patients with the highest ALP tertile had a significantly higher risk of mortality (hazard ratio (HR): 2.26, 95% confidence interval (CI): 1.06–4.82, *p* = .034). Each 10-U/L increase in ALP level was associated with a 4% (HR: 1.04, 95% CI: 1.00–1.08, *p* = .045) higher mortality risk.

**Conclusions:** Higher serum ALP level is associated with increased mortality solely in PD patients with RRF.

## Introduction

Alkaline phosphatase (ALP) is an enzyme catalyzing organic pyrophosphate (a vascular calcification (VC) inhibitor) hydrolysis [1,2], and is released from multiple tissues mainly in the bone, liver, and kidneys [1]. Due to ease of measurement, serum ALP serves to indicate the presence of hepatic and bone diseases reflective of bone turnover and metabolism [3]. Evidence suggests that higher serum ALP levels parallel the risk of mortality in patients without and with chronic kidney disease (CKD), regardless of the dialysis status [4–11]. In the US National Health and Nutrition Examination Survey, researchers have revealed an incremental relationship between higher serum ALP and mortality in the general population [4], while in a study on patients with stages 3 and 4 CKD, it was revealed that elevated serum ALP levels correlated with higher risk of end-stage renal disease (ESRD) and mortality, with a greater effect among patients with higher estimated glomerular filtration rates (eGFRs) [5]. However, a large cohort study identified different findings, showing that the association between serum ALP and mortality stood across a diverse spectrum of renal function, and that this was not affected by the levels of residual renal urea clearance (rCLurea) [8].

It remains unknown whether residual renal function (RRF) influences the association between serum ALP and mortality in peritoneal dialysis (PD) patients. It was hypothesized that high serum ALP levels are predictive of increased mortality risk among PD patients, and that this association would be modified by the RRF status. Hence, a retrospective cohort study was conducted on patients who were followed-up at our PD center for analysis.

## Materials and methods

### Participant enrollment

ESRD patients who chose PD as their first dialysis modality in the PD center of the First Affiliated Hospital, Nanchang University, Jiangxi, China between 1 November 2005 and 28 February 2017 were identified. Inclusion criteria: patients who were ≥18 years old at PD commencement, and whose survival was ≥90 days. Exclusion criteria: patients catheterized in other hospitals, patients transferred from hemodialysis, patients with a history of renal allograft failure, patients with hepatopathy, and patients with unavailable baseline RRF and ALP data. The present study was conducted in compliance with the ethical principles of the Declaration of Helsinki [12]. The Human Ethics Committees of Nanchang University approved the study protocol, and the Institutional Review Board of the First Affiliated Hospital of Nanchang University approved the present study. An informed consent was obtained from each patient.

### Data collection

All patients were followed-up until cessation of PD, death, or 31 May 2017. The demographic data of these patients were recorded, including gender, age, ESRD etiology, and the status of diabetes, hypertension and cardiovascular disease (CVD) at baseline, during the first 1–3 months of PD. Furthermore, their physical and biochemical data were also documented, including body mass index (BMI), blood pressure, hemoglobin, albumin, albumin-corrected calcium, serum phosphorus, serum ALP, intact parathyroid hormone (iPTH), alanine aminotransferase (ALT), aspartate aminotransferase (AST), total Kt/v, total bilirubin (TB), uric acid, triglyceride, high density lipoprotein (HDL)-cholesterol, low density lipoprotein (LDL)-cholesterol, apolipoprotein A1, glycated serum protein, and normalized protein catabolic rate (nPCR) at PD initiation. RRF (mL/min/1.73 m^2^) was estimated from the average of creatinine clearance and urea clearance, and followed-up by adjustments for body surface area. The body surface area was calculated based on the Gehan and George equation [13]:

$$\text{RRF}=\frac{1}{2}\left[\frac{\mathrm{UrineCr}(\mu \text{mol}/L)}{S\mathrm{erumCr}(\mu \text{mol}/L)}\right]+\left[\frac{\mathrm{Urine}\text{Urea}(\text{mmol}/L)}{\mathrm{Serum}\text{Urea}(\text{mmol}/L)}\right]\times \frac{\text{Urine}\;\text{Volume}(\text{mL})}{1440}$$

### Outcome

The primary outcome was all-cause mortality. All patients were followed-up until death, the date of modality switch to hemodialysis, or censored after they received renal transplantation, transferred to other PD center, lost to follow-up, or the end of the present study.

### Statistical analysis

The results were described as frequencies with percentages for categorical variables, means with standard deviations (SDs) for normally distributed continuous variables, or medians with interquartile ranges (IQRs) for non-normally distributed continuous variables. Chi-squared test, one-way analysis of variance (ANOVA), or the Kruskal–Wallis test was used to examine the differences in factors among patients with different RRF statuses.

Patients were categorized according to their RRF status (presence vs. absence). The presence of RRF was defined as having a creatinine clearance of >2 mL/min/1.73 m^2^, according to a prior study [14]. The serum ALP levels were divided into tertiles: RRF group: T1 < 67 U/L, T2 67–92 U/L, and T3 ≥ 92 U/L; non-RRF group, T1 < 60 U/L, T2 60–85 U/L, and T3 ≥ 85 U/L. The characteristics of patients between the RRF and non-RRF groups were compared. Furthermore, the correlation of liver function, bone metabolism and inflammation parameters among patients classified in ALP tertiles were also assessed using the Spearman rank correlation analysis. Cumulative survival was estimated using the Kaplan–Meier curves, and differences in survival probabilities were compared using the Breslow test or log-rank test. The association between serum ALP levels and mortality was examined using Cox proportional hazards models. Variables with a *p* value < .05 in the univariate analyses (data not shown) and those that were clinically important were included in the multivariate model. Then, the relationship between serum ALP levels and outcomes was evaluated using serum ALP, both as a continuous variable and in tertiles. The unadjusted associations were first examined, followed by the adjustment for gender, diabetes, hypertension, CVD, hemoglobin, and albumin. Next, age, serum ALT, serum AST, total Kt/v, albumin-corrected calcium, serum phosphorus, iPTH, TB, HDL-cholesterol, triglyceride, apolipoprotein A1, and nPCR were added into the analysis to determine whether the relationship between ALP and mortality was independent. Covariates with a *p* value < .05 in the univariate analysis, or those that were deemed clinically significant for the subsequent multivariate Cox proportional hazards regression were chosen. The results were described in hazard ratio (HR) and 95% confidence interval (95% CI). All descriptive and multivariate analyses were performed using SPSS version 22.0 (SPSS Inc., Chicago, IL, USA). A *p* value < .05 was considered statistically significant.

## Results

### Patient clinical characteristics

**Figure 1.**
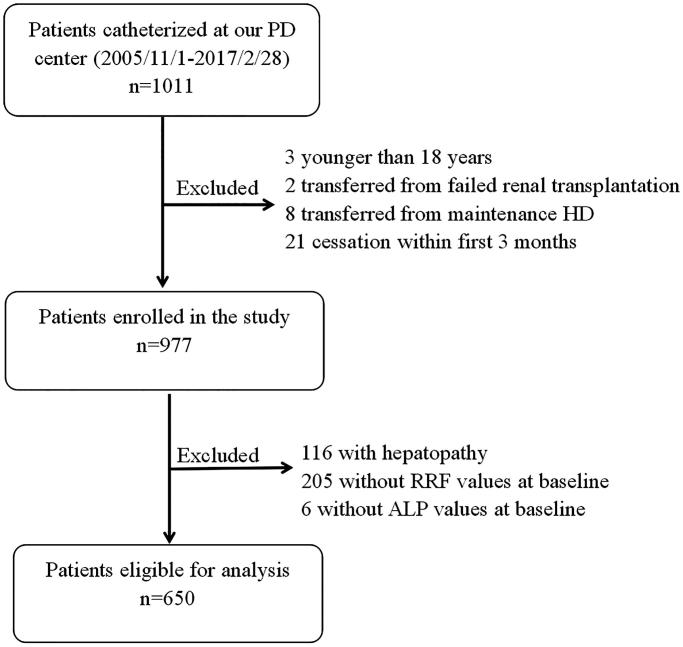
Flowchart showing how patients were selected in the present study.

A total of 1011 incident PD patients were recruited. Among these patients, 34 patients were excluded based on the exclusion criteria: three patients were <18 years old; two patients had a failed allograft, eight patients were transferred from hemodialysis; 21 patients received PD for <3 months. Hence, a total of 977 patients were finally enrolled. Among these patients, 650 patients were free of liver diseases, and had their ALP and RRF measured at baseline (Figure 1).

The mean age of these patients was 49.4 ± 14.0 years old. Furthermore, 56.6% were male, 19.1% had diabetes, and 11.2% had CVD. The most common ESRD etiology in these patients was chronic glomerulonephritis (65.1%), followed by diabetic nephropathy (15.7%), hypertension (12.3%), polycystic kidney (2.0%), obstructive uropathy (2.9%), lupus nephritis (0.6%), hereditary nephropathy (0.4%), and other reasons (0.9%).

### Analysis based on RRF status

The baseline characteristics are presented in Table 1. There were 461 (70.9%) patients in the RRF group, and 189 (29.1%) patients in the non-RRF group. Compared with the non-RRF group, patients in the RRF group had a higher age, hemoglobin, serum albumin, ALP, and nPCR (*p* < .05), but had significantly lower levels of serum phosphorus. However, there were no significant differences between groups in terms of gender, BMI, systolic and diastolic pressure, albumin-corrected calcium levels, iPTH, TB, ALT, AST, triglyceride, HDL-cholesterol, and LDL-cholesterol.

**Table 1.** Baseline features of individuals based on residual renal function.

Variable	All patients (*n*= 650)	With RRF (*n*= 461)	Non-RRF (*n*= 189)	*p* Value
Time of follow-up (months)	39.90 ± 21.92	38.47 ± 20.80	43.39 ± 24.15	<.01
Male gender (%)	368 (56.6)	267 (57.9)	101 (53.4)	.30
Age (years)	49.0 ± 14.0	50.2 ± 13.8	47.6 ± 14.2	.03
Body mass index (kg/m^2^)	22.1 ± 3.4	22.2 ± 3.4	21.7 ± 3.3	.14
Primary kidney disease				
Chronic glomerulonephritis (%)	423 (65.1)	293 (63.6)	130 (68.8)	.20
Diabetic nephropathy (%)	102 (15.7)	76 (16.5)	26 (13.8)	.40
Hypertensive nephropathy (%)	80 (12.3)	59 (12.8)	21 (11.1)	.55
Blood pressure (mmHg)				
Systolic pressure	146.1 ± 26.4	146.1 ± 26.6	146.4 ± 25.9	.90
Diastolic pressure	87.7 ± 16.0	87.3 ± 15.8	88.4 ± 16.3	.44
Hypertension (%)	480 (73.8)	333 (72.2)	147 (77.8)	.14
Diabetes (%)	124 (19.1)	95 (20.6)	29 (15.3)	.12
Cardiovascular disease (%)	73 (11.2)	56 (12.1)	17 (9.0)	.25
Hemoglobin (g/L)	79.4 ± 16.3	80.4 ± 15.9	77.0 ± 17.2	.02
Albumin (g/L)	35.5 ± 5.1	35.9 ± 4.9	34.7 ± 5.5	<.01
Albumin-corrected calcium (mmol/L)	2.07 ± 0.25	2.08 ± 0.12	2.07 ± 0.19	.89
Serum phosphorus (mmol/L)	1.83 ± 0.52	1.78 ± 0.22	1.93 ± 0.45	<.01
Serum alkaline phosphatase (U/L)	74 (59, 98)	76 (60, 102)	71 (55, 89)	.01
iPTH (pmol/L)	206.5 (122.5, 324.8)	206.4 (123.1, 326.1)	210.5 (121.8, 305.8)	.94
Total bilirubin (μmol/L)	3.5 (2.6, 4.7)	3.5 (2.7, 4.6)	3.3 (2.5, 5.0)	.72
ALT (U/L)	13 (9, 21)	13 (9, 21)	12 (9, 21)	.58
AST (U/L)	18 (14, 22)	18 (14, 22.5)	17 (14, 22)	.42
Triglyceride (mmol/L)	1.29 (0.92, 1.80)	1.29 (0.93, 1.84)	1.28 (0.91, 1.74)	.93
HDL-cholesterol (mmol/L)	1.15 ± 0.41	1.14 ± 0.38	1.19 ± 0.47	.12
LDL-cholesterol (mmol/L)	2.48 ± 0.97	2.46 ± 0.95	2.54 ± 1.01	.33
Total Kt/v	2.21 (1.74, 2.74)	2.34 (1.88, 2.86)	1.89 (1.55, 2.42)	<.01
nPCR (g.kg^–1^.d^–1^)	0.70 (0.51, 0.91)	0.71 (0.54, 0.94)	0.64 (0.46, 0.83)	<.01

iPTH: intact parathyroid hormone; ALT: alanine aminotransferase; AST: aspartate aminotransferase; HDL: high density lipoprotein; LDL: low density lipoprotein; nPCR: normalized protein catabolic rate.

### Correlations between serum ALP and other parameters

As shown in Table 2, it was found that there was a significantly positive association between serum ALP levels and albumin, ALT, AST, gamma-glutamyl transferase (GGT), and iPTH (*p* < .05), and a negative association between serum ALP and phosphorus levels (*p* < .05). On the other hand, TB, albumin-corrected calcium, and the neutrophil-to-lymphocyte ratio (N/L) did not correlate with ALP (*p*> .05).

**Table 2.** Correlations between ALP and liver function, bone metabolism and inflammation parameters.

	ALP	ALB	ALT	AST	GGT	TB	CaAlb	P	iPTH
ALB	0.092^b^								
ALT	0.118^a^	–0.130^a^							
AST	0.208^a^	–0.152^a^	0.639^a^						
GGT	0.215^a^	–0.131^a^	0.458^a^	0.348^a^					
TB	–0.045	0.231^a^	0.004	0.056	0.113^a^				
Ca_Alb_	–0.068	–0.139^a^	–0.001	–0.003	0.112^a^	0.100^b^			
P	–0.119^a^	0.092^b^	–0.025	–0.146^a^	–0.052	–0.140^a^	–0.235^a^		
iPTH	0.105^a^	0.132^a^	–0.014	–0.129^a^	–0.133^a^	–0.178^a^	–0.202^a^	0.397^a^	
N/L	–0.034	0.078^b^	0.023	0.019	–0.056	0.015	0.031	–0.072	–0.084^b^

ALB: albumin; ALT: alanine aminotransferase; AST: aspartate aminotransferase; GGT: gamma-glutamyltransferase; TB: total bilirubin; CaAlb: albumin-corrected calcium; N/L: neutrophil to lymphocyte ratio.

a The correlation is significant at the 0.01 level (2-tailed).

b The correlation is significant at the 0.05 level (2-tailed).

### Survival analysis

The median follow-up period was 28 months (IQR: 14–41 months). At study termination, 120 (18.5%) patients died, 94 (14.5%) patients were transferred to hemodialysis, 35 (5.4%) patients received renal transplantation, four (0.6%) patients were transferred to other PD centers, 10 (1.5%) patients discontinued the follow-up, and 387 (59.5%) patients continued to receive follow-ups in our PD center. Among the 120 patients who died, 68 (56.7%) patients were correlated to CVD, nine (7.5%) patients were correlated to infectious disease, one (0.8%) patient was correlated to malignancy, seven (5.8%) patients were correlated to cachexia, 14 (11.7%) patients were correlated to other reasons, and 21 (17.5%) patients were correlated to unknown reasons.

**Figure 2.**
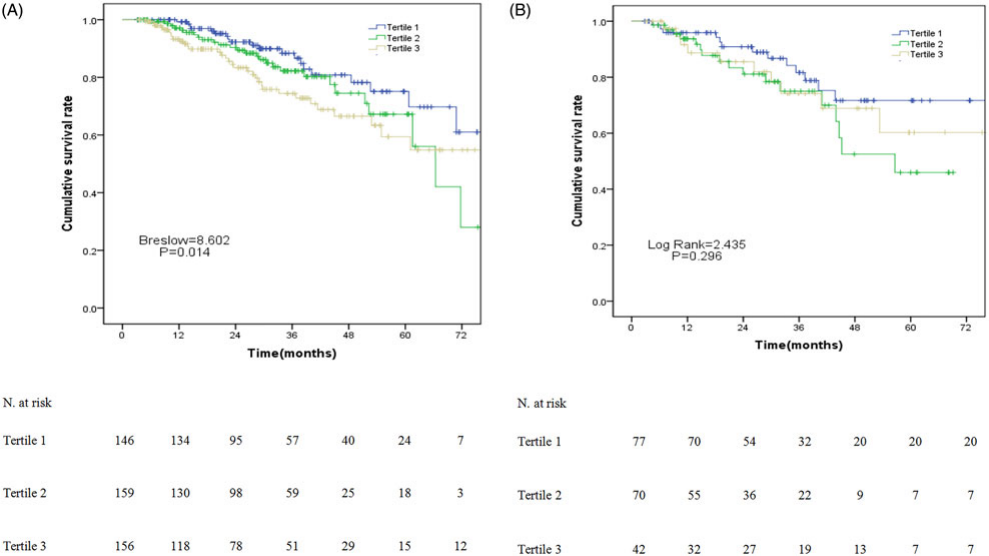
The survival curves for patients stratified by different levels of serum ALP: survival curves for patients with RRF (A); survival curves for patients without RRF (B).

In the RRF group, the 1-, 3-, and 5-year survival rate was 99.3%, 88.4%, and 75.1% in the T1 group, 97.1%, 82.2%, and 67.2% in the T2 group, and 93.3%, 74.4%, and 59.4% in the T3 group, respectively. There were significant differences in survival rate among these three ALP tertiles in the RRF group (*p* = .014, Figure 2(A)). However, there was no significant difference (*p* = .296, Figure 2(B)) among these three ALP tertiles in the non-RRF group.

### Associations between serum ALP levels and mortality stratified based on RRF

The association between serum ALP and mortality in the RRF group was evaluated based on Cox regression analysis. As shown in Table 3, there was a significant association (HR: 2.26, 95% CI: 1.06–4.82; *p* = .034) between the highest level of serum ALP and mortality in the RRF group after adjusting models 2 and 3. After full adjustment, for every 10 U/L of increase in serum ALP level, there would be a 4% increase in HR in all-cause mortality in the non-RRF group (HR: 1.04, 95% CI: 1.00–1.08; *p* = .045). We could not demonstrate an association between serum ALP and mortality in models 1–3 in the non-RRF group.

**Table 3.** Associations between the continuous and tertiles of serum ALP and mortality.

Variables	Model 1^a^		Model 2^b^		Model 3^c^	
	HR (95% CI)	*p* Value	HR (95% CI)	*p* Value	HR (95% CI)	*p* Value
With RRF (*n* = 461)						
Continuous ALP^d^	1.04 (1.00–1.08)	.029	1.04 (1.01–1.08)	.014	1.04(1.00–1.08)	.045
Tertile 1 (<67 U/L)	1.00		1.00		1.00	
Tertile 2 (67–92 U/L)	1.47 (0.82–2.62)	.195	1.54 (0.86–2.78)	.147	2.07 (0.98–4.36)	.056
Tertile 3 (≥92 U/L)	1.93 (1.10–3.36)	.021	1.94 (1.11–3.41)	.020	2.26 (1.06–4.82)	.034
Non-RRF (*n* = 189)						
Continuous ALP^d^	1.02 (0.95–1.09)	.676	1.00 (0.92–1.08)	.938	0.99 (0.89–1.10)	.788
Tertile 1 (<60 U/L)	1.00		1.00		1.00	
Tertile 2 (60–85 U/L)	1.77 (0.86–3.64)	.123	1.01 (0.99–1.03)	.421	1.04 (0.37–2.91)	.945
Tertile 3 (≥85 U/L)	1.41 (0.62–3.22)	.415	1.00 (0.95–1.06)	.963	0.99 (0.35–2.80)	.986

CI: confidence interval; HR: hazard ratio.

a Model 1: unadjusted.

b Model 2: adjusted for gender, diabetes, hypertension, CVD, hemoglobin and albumin.

c Model 3: model 2 adjusted for age, serum ALT, serum AST, total Kt/v, albumin-corrected calcium, serum phosphorus, iPTH, TB, HDL-cholesterol, triglyceride, apolipoprotein A1, and nPCR.

d Per 10 U/L of higher ALP.

## Discussion

This single-center retrospective cohort study revealed that RRF influenced the clinical characteristics of PD patients, and that higher serum ALP levels at dialysis initiation significantly correlated with increased mortality risk solely in PD patients with RRF after adjusting for clinical and biochemical features. To the best of our knowledge, the present study is the first to demonstrate the association between serum ALP and mortality in PD patients with RRF.

Increasing evidence indicates that rising ALP is not only a surrogate for poor bone health, but also a mediator of VC and a predictor of mortality in chronic dialysis patients [1,15,16]. Heterotopic and extra-osseous VC was once considered a process initiated by calcium and phosphate above the solubility threshold, leading to the passive precipitation of apatite mineral in vessels and soft tissues. This theory evolved after more *in vitro* and *in vivo* models of VC have become available [1]. Animal studies that focused on tissue-nonspecific alkaline phosphatase (TNAP) in VC revealed that TNAP is upregulated in vessels of uremic rats. This contributes to the hydrolysis and inactivation of inorganic pyrophosphate, which is a potent inhibitor of hydroxyapatite crystal growth and VC. Animals with genetically ablated TNAP exhibited less VC, while levamisole, a nonspecific ALP inhibitor, ameliorated the hydrolysis rate of pyrophosphate in the aortas of uremic rats [16].

Elevated ALP predicted a higher mortality among the general population, and populations with mild to severe CKD or transplant recipients [4–6,8,11,17–19]. Abramowitz et al. [6] found that in a multi-ethnic inner-city cohort with an eGFR of ≥60 mL/min/1.73 m^2^, having an ALP level of >104 U/L was associated with 65% higher risk of mortality. Furthermore, high-normal serum ALP levels also increase the risk of CVD-related hospitalization, when compared to others. Furthermore, data from the African-American Study of Kidney Disease and Hypertension (AASK) database revealed that the doubling of the baseline ALP level was associated with a 55% increase in all-cause mortality rate in CKD stages 3 and 4. However, there was no association noted between ALP level and the composite of death, dialysis, or GFR events [17]. Taliercio et al. [5] found that in 28,678 patients with CKD stages 3 and 4, for every 42.7 U/L (onefold SD) increase in ALP level, there was an associated 15% and 16% higher risk of ESRD and mortality, respectively. This study also revealed an interaction between eGFR and ALP level, in terms of the influences on mortality, presenting as a greater hazard of ALP levels among those with higher eGFRs. However, Wang et al. [8] did not observe a consistent association between higher ALP and mortality across different rCLurea levels. Patients with greater RRF consistently had better survival rates, when compared to patients with the same level of ALP. This highlights the importance of RRF in the dialysis population. In this sense, existing studies have shown that RRF is closely associated with the prognosis of CKD/ESRD patients, and that there might be an interaction between RRF and ALP levels. Regrettably, none of these further elaborates the mechanism by which RRF affects the association of ALP and patient death.

RRF, the remaining function of kidneys among patients with ESRD [20], confers multiple benefits to patients under chronic dialysis, and these benefits include permitting a lower ultrafiltration volume, less intradialytic hypotension, a lower prevalence of anemia and a decelerated course of malnutrition and inflammation, lower erythropoietin resistance, and ventricular hypertrophy [21–23]. RRF levels are also inversely associated with the probability of VC [24]. Evidence suggests that higher RRF is independently associated with better survival in chronic dialysis patients [25–27].

In the present study, it was found that the correlation between ALP and mortality in PD patients differed based on RRF status, but the underlying reason remains unclear. The putative link among RRF, oxidative stress and atherosclerosis may be one plausible explanation. By measuring plasma advanced oxidation protein products and serum pentosidine, Furuya et al. [28] discovered an inverse relationship between RRF and oxidative substances in PD patients. Oxidative stress is associated with endothelial dysfunction [29], malnutrition [30] and higher cardiovascular mortality [31]. In addition, *in vitro* experiments that used vascular and bone cells revealed that oxidative stress can be a strong inducer of ALP, and promotes the transition of vascular cells into calcifying cells [32]. On the other hand, ALP is also part of the defense molecule that protects the host against bacterial agents, which can be potently induced by IL-6, TNF-α and bacterial lipopolysaccharide, leading to inflammation and higher oxidative stress [33]. Consequently, the investigators consider that RRF may interact with ALP with regard to clinical outcomes.

Arteriosclerosis and atherosclerosis are frequently accompanied by preclinical arterial changes in PD patients [34]. Arterial stiffness increase often results from multiple factors. Among these factors, poor RRF is an important factor. Rroji et al. [35] revealed that RRF is an independent risk factor for atherosclerosis. Interestingly, atherosclerosis *per se* is also known as an inflammatory process [34], and a retrospective study suggested that inflammation independently predicted more rapid loss of RRF in PD patients [36]. Bench work repeatedly revealed that pro-inflammatory cytokines stimulated the production of active vitamin D in vascular smooth muscle cells (VSMCs), and promoted the calcification of VSMCs by upregulating ALP expression [37,38]. This may also be responsible for the relationship between poor RRF and increasing mortality, as well as CVD, in these patients.

RRF contributes to erythropoietin and active vitamin D3 production, and improves hematopoiesis, nutrition and the maintenance of calcium and phosphorus [39]. In the present study, hemoglobin levels were higher in the RRF group than in the non-RRF group, while serum phosphate was higher in the non-RRF group. These present findings support the presence of the influence of RRF in the endocrine system. It was also found that higher serum ALP increased the risk of mortality solely in PD patients with RRF, and not in PD patients without RRF. Several reasons may be responsible for the discrepancy in the relationship between serum ALP and mortality between patients with and without RRF. First, ALP hydrolyzes pyrophosphate and increases VC. RRF levels are inversely associated with the probability of VC [24], which contributes to a higher mortality among PD patients [40]. Second, patients in the non-RRF group tended to have worse nutritional and inflammatory parameters, with a higher prevalence of ventricular hypertrophy and other comorbidities, and all of which possess a close association with the increased mortality in PD patients. Finally, it is also plausible that overall mortality is more likely to be affected by RRF than by ALP. Future studies are needed to explore the reasons behind the relationship between ALP and RRF in these patients.

There were some limitations in the present study. First, the present study was single-center in nature, and a center-specific effect was possible. Second, its retrospective nature did not permit the identification of causal relationships. Third, bone-specific ALP was a more sensitive and specific marker for bone histology, when compared to iPTH and ALP. However, bone-specific ALP was not routinely measured due to the availability and cost associated with the assay. Finally, due to sample size restrictions, factors associated with higher mortality were not comprehensively adjusted. Furthermore, the effect of residual confounding could not be completely excluded.

## Conclusions

The present study demonstrated that the relationship between serum ALP and mortality could be modified by RRF among PD patients, and that higher serum ALP levels are correlated with increased mortality solely in patients with RRF.
